# Urine and Serum MicroRNA-1 as Novel Biomarkers for Myocardial Injury in Open-Heart Surgeries with Cardiopulmonary Bypass

**DOI:** 10.1371/journal.pone.0062245

**Published:** 2013-04-22

**Authors:** Xian Zhou, Anqiong Mao, Xiaobin Wang, Xiaoxia Duan, Yi Yao, Chunxiang Zhang

**Affiliations:** 1 Department of Internal Medicine and Anesthesiology and Critical Care Medicine, Affiliated Hospital of Luzhou Medical College, Luzhou, Sichuan Province, China; 2 Rush University Cardiovascular Research Center and Department of Pharmacology, Rush Medical College, Rush University, Chicago, Illinois, United States of America; Northwestern University, United States of America

## Abstract

MicroRNA-1 (miR-1) is a cardio-specific/enriched microRNA. Our recent studies have revealed that serum and urine miR-1 could be a novel sensitive biomarker for acute myocardial infarction. Open-heart surgeries with cardiopulmonary bypass (CPB) are often accompanied with surgery injury and CPB-associated injury on the hearts. However, the association of miR-1 and these intra-operative and post-operative cardiac injures is unknown. The objective of this study was to test the hypothesis that urine and serum miR-1 might be a novel biomarker for myocardial injuries in open-heart surgeries with CPB. Serum and urine miR-1 levels in 20 patients with elective mitral valve surgery were measured at pre-surgery, pre-CPB, 60 min post-CBP, and 24h post-CBP. Serum cardiac troponin-I (cTnI) was used as a positive control biomarker for cardiac injury. Compared with these in pre-operative and pre-CPB groups, the levels of miR-1 in serum and urine from patients after open-heart surgeries and CPB were significant increased at all observed time points. A similar pattern of serum cTnI levels and their strong positive correlation with miR-1 levels were identified in these patients. The results suggest that serum and urine miR-1 may be a novel sensitive biomarker for myocardial injury in open-heart surgeries with CPB.

## Introduction

MicroRNAs (miRNAs) are a novel class of small, endogenous non-coding RNA molecules with strong biological functions in normal development, physiology and disease states, including cardiovascular disease [1].

Tissue- and cell-specific expression is one important characteristic of miRNA expression. Indeed, one miRNA may be highly expressed in one tissue or one cell, but has no or very low expression in other tissues or cells. For example, miR-1 is reported to be a muscle- or heart-enriched miRNA [2], whereas miR-145 is a vascular smooth muscle cell-specific miRNA, as described in our recent article [3]. The tissue-specific miRNA expression and expression signatures of miRNAs have provided a great diagnostic opportunity for diverse human diseases.

However, unlike tumors, human heart samples are not easy to obtain in clinical practice. Interesting, recent studies from us and other groups have demonstrated that the diseased hearts can release cardiac miRNAs into the circulating blood [4], [5]. In contrast with our original hypothesis, cell-free blood miRNAs are relatively stable due to binding with other materials such as exosomes [4], [5]. More recently, we have identified that the heart-released miRNAs such as miR-1 could enter into animal and human urine [6]. Both serum and urine miR-1 could be used as a novel sensitive biomarker for heart damages.

Open-heart surgeries with cardiopulmonary bypass (CPB) are widely used to treat many heart diseases. However, the surgeries themselves may have intra-operative injury on hearts [7]. CPB is an essential component of open-heart surgeries. Recent studies have revealed that CPB is associated with multiple organ injuries including heart, brain, lung and kidney damages both intra-operatively and post-operatively, although the molecular mechanisms responsible for them are still unclear [8. 9]. Moreover, these intra-operative and post- operative cardiac injures are associated very well with the operation-related complications and deaths. Establishing of sensitive biomarkers for the heart damages induced by the open-heart surgeries and CPB is thus critical in selecting the optional treatments in these patients.

Based on our recent translational studies [4. 6], we hypothesize that urine and serum miR-1 might be a novel biomarker for myocardial injury in the open-heart surgeries with CPB. In this study, we are trying to test our hypothesis by determining the serum and urine miR-1 levels in 20 patients with elective mitral valve surgeries and CPB.

## Methods

### Patients

After obtaining the written informed consents, twenty adult patients of either sex undergoing elective mitral valve surgery with the aid of CPB were included in this study. Patients with diabetes mellitus; hepatic, renal, or neurological dysfunction; recent myocardial infarction (MI); unstable angina; coagulopathy; left ventricular ejection fraction <45%; preoperative congestive heart failure (CHF); preoperative hemodynamically unstable arrhythmias and those undergoing concomitant valve surgery were excluded from the study.

### Ethics Statement

This study was approved by the Ethics Committee of Luzhou Medical College Hospital.

### Anesthesia, CPB and the Open-heart Surgeries

Anesthesia was induced with intravenous midazolam (1–2 mg), etomidate (0.3 mg.kg^−1^), and fentanyl (2–5 µg.kg^−1^) and was maintained with oxygen (100%) and supplemental doses of intravenous fentanyl, midazolam, vecuronium and isoflurane. The lungs were mechanically ventilated to maintain normocapnia and a pH of 7.35–7.45. The right internal jugular vein was cannulated with 7.5 Fr triple lumen central venous catheter for fluid, vasopressor/inotrope administration and central venous pressure measurement. In addition, pulse oximetry, nasopharyngeal temperature, five lead ECG and urine output were monitored. Volume replacement was done with hydroxyl ethyl starch (Voluven 6% Fresenius Kabi, Germany) or Ringer’s lactate as appropriate to maintain the CVP at 5–13 mmHg. Aortic and atrial cannulations were done after systemic heparin with intravenous heparin 400 IU kg^−1^, to achieve an activated clotting time of >500 sec. All patients underwent cardiac surgery with a standard CPB protocol under moderate hypothermia at 29–31°C. Pump flow rates and perfusion pressures were maintained at 2.3–2.7 L/min/m2 and 50–70 mmHg, respectively. α-stat strategy was used for blood gas management and blood sugar was maintained between 100 and 200 mg/dl during CPB. A hematocrit ≥20% was maintained.

### Clinical Characteristics

Heart rate (HR), mean arterial pressure (MAP), CVP and cardiac index (CI) were recorded at pre-surgery (T_0_), pre-CPB (T_1_), 60 min post-CPB (T_2_) and 24 hours post-CPB (T_3_). CPB time, cross-clamp time and inotropic requirement were also recorded by nurses who were unaware of the study design.

### Blood and Urine Sample Collection, miRNA Isolation, and Measurements of miR-1 and cTnI

Blood and urine samples were obtained at pre-surgery (T_0_), pre-CPB (T1), 60 min post-CPB (T2) and 24 hours post-CBP (T3). Blood gas analysis was done and hematocrit was recorded at each time point. Serum and urine samples were stored at −20°C until analysis. Serum cTnI levels were measured by Abbott automatic biochemistry analyzer. Serum and urine miR-1 levels were measured using the qRT-PCR based solution miRNA quantitative kit developed by our group [4], [5].

### Statistical Analysis

All data is presented as mean ± SD. For relative gene expression, the mean value of the control group was defined as 1. Differences in miRNA levels were analyzed using one-way ANOVA with Bonferroni’s multiple comparisons post hoc test. Linear regression analysis was used to determine the relationship between serum cTnI and serum or urine miR-1. A p value<0.05 was considered significant.

## Results

### The Characteristics of the Patients

Distribution of sex, age, weight, type of heart diseases, pre-operative cardiac functional grading, preoperative serum cTnI, preoperative artery blood gas analysis and pre-operative cardiac function were shown in the Table 1. There were no operation-related deaths or serious complications in these patients.

**Table 1:** Characteristics of the patients.

Age (Years)			46.76±15.08
Sex			
	Female (n)		8
	Male (n)		12
Weight (kg)			56.56±12.21
Mitral valve disease			
	Stenosis (n)		3
	Regurgitation (n)		1
	Stenosis and Regurgitation (n)		16
Etiology			
	Rheumatic heart disease (n)		20
Preoperative indices			
	Ejection Fraction (%)		62.78±8.26
	Cardiac Function (NYHA)		
		II (n)	7
		III (n)	13
Plasma cTnI (ng/ml)		Nomal (n)	20
Artery blood gas analysis		normal (n)	20
cardiopulmonary bypass time (min)			86.6±9.6
Cross-clamp time (min)			48.2±7.2
Cumulative postoperative dosage of dobutamine (mg)			438.6±126.9
Intensive care stay (h)			58.1±6.6
Postoperative hospital stay (days)			11.5±2.5

### Open-heart Surgeries with CBP are Accompanied with Acute Cardiac Injury as shown by the Increased Levels of Serum cTnI

**Figure 1:**
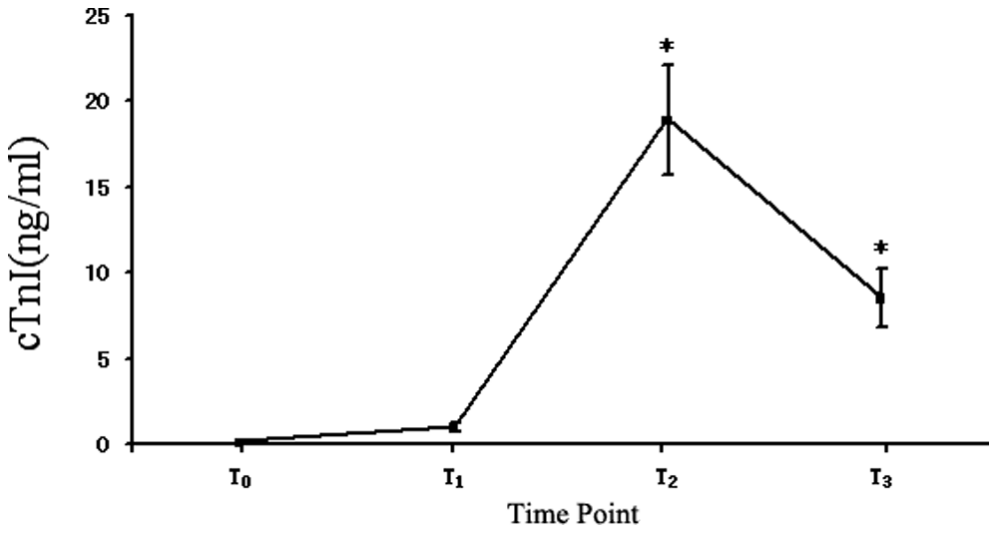
The serum levels of cardiac troponin (cTnI) in patients with the open-heart surgeries and CPB. n = 20, *P<0.01 vs T_0_. T_0_, before surgery (pre-surgery); T_1_, before CPB (pre-CPB); T_2_, 60 min after CPB (post-CPB); and T_3_, 24 hours after CPB (post-CPB).

As shown in Figure 1, the serum levels of cTnI were significantly increased in patients after surgeries and CBP, compared with those in pre-surgery and pre-CPB controls. The serum cTnI levels were peaked at 1 h after CBP, but were still higher than those in controls at 24 h after CBP.

### Open-heart Surgeries with CBP Increase Serum and Urine Levels of miR-1

**Figure 2:**
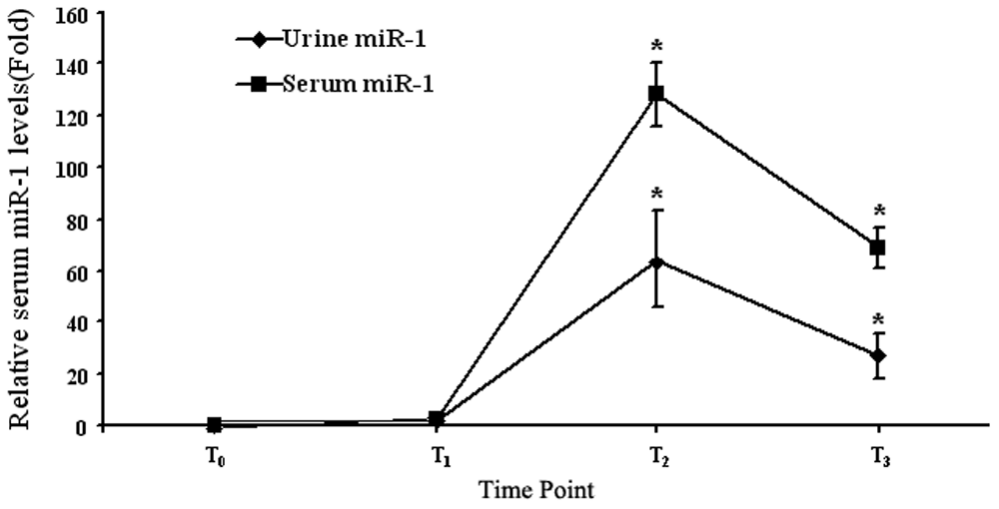
The serum and urine miR-1 levels in patients with the open-heart surgeries and CPB. n = 20, *P<0.01 vs T_0_. T_0_, before surgery (pre-surgery); T_1_, before CPB (pre-CPB); T_2_, 60 min after CPB (post-CPB); and T_3_, 24 hours after CPB (post-CPB).

As shown in Figure 2, there was no difference in miR-1 levels both in serum and urine samples between pre-surgery (T_0_) and pre-CPB (T1). However, there was a quickly increase in miR-1 levels in patients after heart opening and CPB. The miR-1 levels were peaked at 1 h after CBP, but were still higher than those in controls at 24 h after CBP. Clearly, the changes of miR-1 levels shared the similar pattern with those of serum cTnI.

### Correlation of miR-1 Levels and cTnI Levels

**Figure 3:**
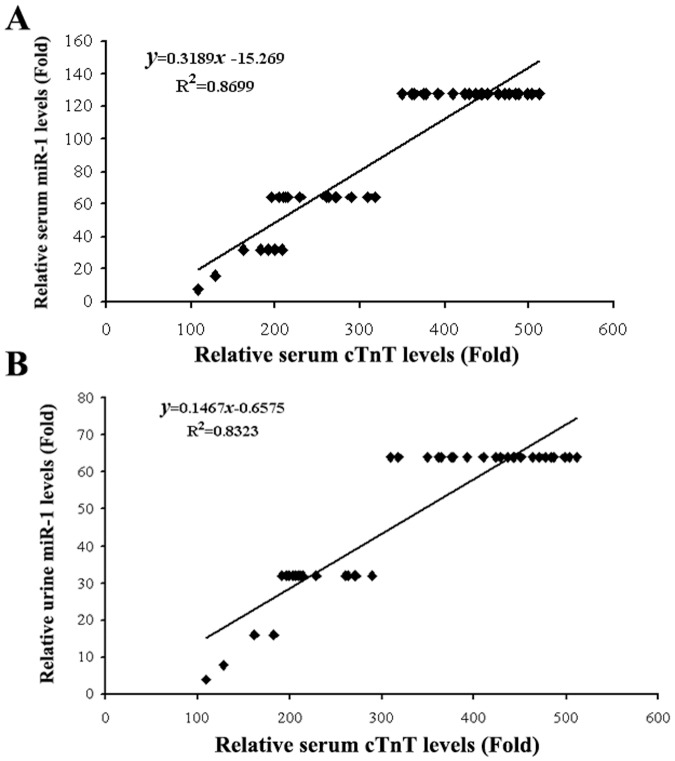
The relationship between miR-1 and cTnI levels. A). The relationship between serum miR-1 and serum cTnI levels in patients with the open-heart surgeries and CPB. A positive correlation was demonstrated between the two variables. n = 20, r = 0.93; P<0.05. B). The relationship between urine miR-1 and serum relative cTnI levels. A positive correlation was demonstrated between the two variables. n = 20, r = 0.93; P<0.05.

To determine the potential association between miR-1 and cTnI, the serum cTnI levels, and miR-1 levels in both serum and urine were analyzed by linear regression. As shown in Figure 3, strong positive correlations were found between serum cTnI levels and serum miR-1, as well as urine miR-1 levels, in patients with the open-heart surgeries and CBP.

## Discussion

The currently used biomarkers for acute myocardial infarction (AMI), unstable angina and peri-operative myocardial injury are blood creatinine phosphokinase-muscle band (CPK-MB), troponin-T (TnT), and troponin I (TnI) [10. 11]. In the current study, we find that the serum levels of cTnI are significantly increased in patients with the open-heart surgeries and CBP. The result is consistent with the previous reports, suggesting that the open-heart surgeries with CPB are accompanied with the cardiac injury.

The recent studies from us and other groups suggest that the blood miR-1 may represent a novel circulating biomarker for AMI [4], [5]. However, up to date, there is no study to determine the blood miR-1 levels in open-heart surgeries. Here we find that serum miR-1 levels are significantly increased in patients after the open-heart surgeries and CBP. Thus, the blood miR-1 levels may reflect the potential surgery-related cardiac injuries.

Currently, there are no established urine biomarkers for AMI, because the blood protein biomarkers such as TnT are difficult to be filtered into urine. However, our recent study has discovered that the heart-released miR-1 can enter both rat and human urine, and the urine miR-1 may service as a novel biomarker for AMI [6]. This finding is further verified by another independent group, in which urine miR-1 is increased in both pig and human urine after AMI [12]. In the current study, we are excited to find that urine miR-1 is also increased in patients with the open-heart surgeries and CBP.

Interestingly, the changes of serum and urine miR-1 levels shared the similar pattern with those of serum cTnI. We further identified that there are strong positive correlations between serum cTnI levels and serum miR-1 levels, as well as urine miR-1 levels, in patients with the open-heart surgeries and CBP. The results suggest that both urine and serum microRNA-1 may be a novel biomarker for myocardial injury in open-heart surgeries with CBP.

In addition to a direct cardiac injury induced by heart opening, CBP also has an injury effect on heart. Indeed, CPB is associated with multiple organ injuries including heart, brain, lung and kidney damages, although the molecular mechanisms responsible for them are still unclear [8], [9]. The current study design cannot separate the heart-open-induced and the CBP-induced increase in miR-1 levels. However, we think both injuries may be involved in the increased miR-1 levels. Clearly, additional studies are needed to evaluate individual impact from the two different injures.

In summary, in this study, we identified that both serum and urine miR-1 could be a novel biomarker for cardiac injury in open-heart surgeries with CBP. These miR-1 levels may be useful in selecting different treatments in patients after the open-heart surgeries.
